# An IL13Rα2 peptide exhibits therapeutic activity against metastatic colorectal cancer

**DOI:** 10.1038/s41416-018-0259-7

**Published:** 2018-10-15

**Authors:** Rubén A. Bartolomé, Marta Jaén, J. Ignacio Casal

**Affiliations:** Department of Molecular Biomedicine, Centro de Investigaciones Biológicas, CSIC, Ramiro de Maeztu 9, 28039 Madrid, Spain

**Keywords:** Colorectal cancer, Metastasis, Targeted therapies

## Abstract

**Background:**

Interleukin 13 receptor α2 (IL13Rα2) is overexpressed in metastatic colorectal cancer. Here, we have developed novel strategies to block IL-13 binding to IL13Rα2 in order to reduce metastatic spread.

**Methods:**

Synthetic IL13Rα2 D1 peptide (GSETWKTIITKN) was tested for the inhibition of IL-13 binding to IL13Rα2 using ELISA and different cellular assays. Peptide blocking effects on different cell signalling mediators were determined by western blot. An enantiomer version of the peptide (D-D1) was prepared to avoid proteolytic digestion. Nude mice were used for tumour growth and survival analysis after treatment with IL13Rα2 peptides.

**Results:**

IL13Rα2 D1 peptide inhibited migration, invasion, and proliferation in metastatic colorectal and glioblastoma cancer cells treated with IL-13. Residues ^82^K, ^83^T, ^85^I and ^86^T were essential for blocking IL-13. IL13Rα2 peptide abolished ligand-mediated receptor internalisation and degradation, and substantially decreased IL-13 signalling capacity through IL13Rα2 to activate the FAK, PI3K/AKT and Src pathways as well as MT1-MMP expression. In addition, D1 significantly inhibited IL-13-mediated STAT6 activation through IL13Rα1. Nude mice treated with the enantiomer D-D1 peptide showed a remarkable survival increase.

**Conclusions:**

We propose that the D-D1 peptide from IL13Rα2 represents a promising therapeutic agent to inhibit metastatic progression in colorectal cancer and, likely, other solid tumours.

## Background

Recently, we described a prometastatic role for interleukin 13 (IL-13) in colorectal cancer mediated through the interleukin 13 receptor α2 (IL13Rα2).^1^ Both molecules, IL13 and IL13Rα2, are expressed in tumoural tissue samples.^1^ IL13Rα2 has been classified as a cancer/testis-like tumour antigen^2^ that is upregulated following activation of EGFR and mutant EGFRvIII in cancer cells.^3^ In colorectal cancer, IL13Rα2 expression was mainly associated to late stage tumours and lower overall survival in colorectal cancer patients.^1^ IL13Rα2 is overexpressed in a variety of human tumour types such as glioblastoma, renal cell carcinoma, pancreatic, melanoma, head and neck, mesothelioma and ovarian, where it has been proposed as biomarker and potential therapeutic target.^4–11^ In glioblastoma, IL13Rα2 has been associated with a more aggressive mesenchymal gene expression signature and a poorer patient prognosis.^12^

IL13Rα2 is structurally different from the classical IL13Rα1 receptor. IL13Rα2 expression is specific for cancer cells while IL13Rα1 is not. IL13Rα2 is present in two forms: a membrane signalling receptor and a soluble form with no signalling activity.^13^ The binding of IL-13 to IL13Rα2 triggers different cellular pathways, STAT6-independent, to promote migration, invasion and survival of colorectal cancer metastatic cells.^1^ Initially, the strong binding affinity of IL-13 to IL13Rα2 was interpreted as a way to sequester IL-13 and provoke its downregulation.^14^ However, the decoy concept does not explain IL13Rα2 overexpression in cancer cells and its signalling capacity. In metastatic colorectal cancer cells, IL13Rα2 signalling is mediated through a scaffold protein, FAM120A, which activates FAK and PI3K pathways and, indirectly, Src.^15^ In addition, IL13Rα2 associates with multiple intracellular traffic proteins and its recycling is tightly controlled to regulate the surface membrane expression levels and the amount of free receptor.

Targeting IL13Rα2 for cancer therapy has been the subject of numerous studies and different strategies, including immunotoxins, DNA vaccines and specific monoclonal antibodies (see ref. ^16^ for a review). Some trials involved the use of an IL-13 immunotoxin, containing a truncated version of *Pseudomonas* exotoxin, highly cytotoxic to renal cancer cells and other human solid tumours.^17^ Indeed, IL-13 immunotoxin has been used in a Phase III clinical trial with glioblastoma patients, showing small but significant effects on survival.^18^ These IL-13 immunotoxin-based strategies might bind indistinctly IL13Rα2 or IL13Rα1 receptors. Another strategy made use of a high-affinity antibody to IL13Rα2, which led to a modest increase in the survival of mice intracranially implanted with human glioma xenografts.^19^ In addition, IL-13-neutralising monoclonal antibodies (i.e. Tralokinumab^™^) with the capacity to prevent IL-13 binding to both receptors, IL13Rα2 and IL13Rα1, have been described.^20^ Recently, a chimeric antigen receptor T-cell therapy targeting IL13Rα2 caused a substantial regression of glioblastoma in a patient.^21^ Finally, using phage-display technology, Pandya et al. identified a peptide with capacity to bind IL13Rα2 and glioblastoma cells.^22^ When radiolabeled, this peptide caused a significant increase in cellular cytotoxicity and mouse survival.^23^ However, this peptide did not match with any IL-13 sequence and its binding seems to be IL-13 independent.

Since no clinical trials have been developed for targeting IL13Rα2 in metastatic colorectal cancer, we propose a novel strategy based on blocking the IL-13/IL13Rα2 signalling axis with IL13Rα2 peptides to inhibit their prometastatic functions. For peptide design, we used the structural information available for the IL-13/IL13Rα2 binding complex.^24^ The structure of the high-affinity complex between IL-13 and IL13Rα2 was recently elucidated and involves three IL13Rα2 domains: the N-terminal domain (D1) and two fibronectin-like domains (D2 and D3), including in total two major binding sites, II and III.^24^ The selected region contains the linear sequence ^81^WKTIITKN^88^, which is highly conserved in mammalians, suggesting a key role in the binding to IL-13.

Here, we provide evidence that a 12 amino acid-long peptide containing the ^81^WKTIITKN^88^ conserved sequence from the IL13Rα2 binding site might be effective as therapeutic agent in metastatic colorectal cancer. The blocking peptide inhibited the signalling pathways in metastatic cells based on IL13Rα2 and, at a lower extent, IL13Rα1. A D-enantiomer version of the peptide demonstrated a superior “in vivo” stability and significantly increased mice survival by suppressing metastatic colonisation.

## Methods

### Cell lines, siRNAs, antibodies, and peptides

Highly metastatic KM12SM and nonmetastatic KM12C human colorectal cancer cells were obtained directly from Dr. Fidler (MD Anderson Cancer Center, Houston, TX). T98MG, U87MG and U118MG glioblastoma cell lines were provided by Dr. G Velasco (Universidad Complutense de Madrid, Spain). U251MG glioblastoma cell line was a gift from Dr. I Balyasnikova (Northwestern University, Chicago, USA). Cells were authenticated by short tandem repeat analysis and regularly tested for mycoplasma contamination. Human SW480, SW620, HT-29 and mouse CT-26 colon cancer cell lines were purchased from the ATCC and passaged fewer than 6 months after purchase for all the experiments. All cell lines were cultured in DMEM (Invitrogen, Spain) containing 10% FCS (Invitrogen, Carlsbad, CA, USA) and antibiotics at 37 °C in a 5% CO_2_ humidified atmosphere. For transient transfections, siRNAs targeting specifically IL13Rα1 (SASI_Hs01_00041228, Sigma-Aldrich, Spain), IL13Rα2 (SASI_Hs01_00156457, Sigma-Aldrich) or Control (5′-AUUGUAUGCGAUCGCAGACdTdT-3′) were transfected with JetPrime (Polyplus Transfection, Illkirch, France) according to the manufacturer’s instructions.

IL13Rα2 (2K8), RhoGDIα (G-2), AKT (5C10), MMP9 and FAK (A-17) antibodies were purchased from Santa Cruz Biotechnology (Dallas, TX, USA). Antibodies against pY397-FAK (#611722) and Src (AF3389) were from BD Biosciences (San Jose, CA, USA) and R&D Systems (Minneapolis, MN, USA), respectively. Anti-IL13Rα1 (ab79277) was from Abcam (Cambridge, UK). Antibodies against phospho-Src (#2101), STAT6 (#9362), phospho-STAT6 (#9364), phospho-Akt (#4060), ERK1/2 (L24F12) and phospho-ERK1/2 (#9101) were from Cell Signaling Technology (Danvers, MA, USA). MT1-MMP antibody was a kind gift of Dr. A. García-Arroyo (CIB-CSIC).

Recombinant human IL-13 was from PeproTech (London, UK). IL-13 mutants were obtained from Protein Alternatives SL (Tres Cantos, Madrid, Spain). IL13Rα2 peptides (D1: GSETWKTIITKN; D1S: WKTIITKN; Nter: FEIVDPGY; Cter WSIPLGPI; D1-1A: WATIITKN; D1-2A WKAIITKN; D1-3A WKTAITKN; D1-4A WKTIATKN; D1-5A WKTIIAKN; D1-6A WKTIITAN) and IL13Rα1 peptide (KQDKKIAPE) were synthesised using solid phase chemistry with a Focus XC instrument (AAPPtec). The enantiomer peptide (D-D1) (GSETWKTIITKN) was purchased from Proteogenix (Schiltigheim, France). Peptides were used at 1 μg/mL in the different experiments.

### Competition ELISA

Microtiter plates (Maxisorp, Nunc) (Thermo Fisher Scientific, Waltham, MA, USA) were coated overnight with 3 μg/mL of the purified IL13Rα2 ectodomain (Protein Alternatives SL). After washing three times with PBS, plates were blocked with 3% skimmed milk in PBS for 2 h at room temperature. Then, IL-13 (0.3 μg/mL) and the D1 peptides at different concentrations (1–50 µg/ml) were added to the plates, which were incubated for 1.5 h at room temperature. After washing, peroxidase-labelled anti-IL-13 antibody (Abexxa, Cambridge, UK) (0.4 μg/mL) was added for 1.5 h at room temperature. Colour was developed with 3, 3′, 5, 5′-tetramethylbenzidine substrate (Sigma-Aldrich, Saint Louis, MO, USA). The reaction was stopped with 1 M HCl, and absorption measured at 450 nm.

### Proliferation assays

Cancer cells were seeded at 1×10^4^ cells/well on 96-well plates and were incubated for 48 h at 37 °C in DMEM with 0.5% serum in presence of IL-13 (10 ng/mL) and the indicated peptides, followed by 1 h incubation with Thyazolyl Blue Tretrazolium Bromide (MTT) (0.6 mg/mL) (Sigma-Aldrich). Cell proliferation was determined by absorbance at 560 nm and comparison with control cells collected ab initium.

### Cell adhesion assays

Cancer cells were starved for 3 h, labelled with BCECF-AM (Molecular Probes, Eugene, OR, USA), detached with EDTA/PBS and incubated in DMEM in the presence of IL-13 and the indicated peptides for 10 min at 37 °C. Then, 6×10^4^ cells in 100 μL were added to 96-well plates previously coated with Matrigel (0.4 μg/mm^2^) (BD Biosciences), and the plates were incubated for 25 min at 37 °C. Subsequently, nonadhered cells were removed by three washes with DMEM. Bound cells were lysed with 1% SDS in PBS, and the extent of the adhesion was quantified using a fluorescence analyser (POLARstar Galaxy, BMG Labtech, Ortenberg, Germany).

### Wound healing and invasion

Cancer cells were cultured to confluence in Matrigel-coated plates (0.4 μg/mm^2^), and a 1-mm-wide wound was made across the monolayer. Cells were incubated in serum-free medium with or without IL-13 (10 ng/mL), in the presence or absence of the indicated peptides. Pictures for quantification were taken immediately and after 24 h at 37 °C. Effective migration speed was calculated as the distance covered by cells in 24 h in each side of the wound.

For invasion assays, 6×10^4^ cells were loaded onto 8 mm pore-size filters coated with 35 mL of Matrigel (1:3 dilution; BD Biosciences) in Transwell plates (Sigma-Aldrich) in presence of the indicated peptides. The lower compartment of the invasion chamber contained IL-13 (10 ng/mL). After 48 h, noninvading cells were removed from the upper surface of the filter, and cells that migrated through the filter were fixed with 4% paraformaldehyde (Sigma-Aldrich), stained with crystal violet and counted under a microscope.

### Flow cytometry

For flow cytometry, cells were detached with 2 mm EDTA in PBS, incubated at 4 °C with primary antibodies for 30 min, washed and incubated with Alexa488 labelled-secondary antibodies (Agilent, Santa Clara, CA, USA). Fluorescence was analysed in a Coulter Epics XL cytofluorometer (Beckman Coulter, Brea, CA, USA). Mean fluorescence intensity of 10,000 analysed cells is shown for each cell type. For IL13Rα2 internalisation assays, KM12SM and U251 cells were exposed to IL13 and/or D1 peptide for 1 h and subjected to flow cytometry to detect IL13Rα2 in cell surface. Expression of this receptor is shown as percentage of the nonexposed cells, mean fluorescence intensity was 1.01.

### Cell signalling analysis

Cancer cells were incubated for 3 h in serum-free DMEM, detached with 2 mM EDTA, washed and treated with IL-13 (10 ng/mL) at different times with or without D1 peptide. Then, cells were treated with lysis buffer (1% Igepal, 50 mM Tris-HCl, 100 mM NaCl, 2 mM MgCl_2_, 10% glycerol, protease inhibitors (Complete Mini, Roche, Basel, Switzerland) and phosphatase inhibitor cocktails 2 and 3 (Sigma-Aldrich)). Protein extracts (60 µg) were separated in SDS-PAGE and transferred to nitrocellulose membranes, which were incubated with the indicated primary antibodies, washed and incubated with HRP-conjugated secondary antibodies (Sigma-Aldrich). Membranes were revealed using SuperSignal West Pico chemiluminescent Substrate (Thermo Scientific).

### Zymography assays

Cancer cells were incubated in serum-free medium for 24 h with or without IL-13 (10 ng/mL), in the presence or absence of D1 peptide. Collected conditioned media were concentrated ten times using Vivaspin 15R (Sartorius, Stonehouse, UK). Fifteen microlitres of sample were mixed with Laemmli buffer and resolved under nonreducing conditions on SDS-PAGE gels embedded with 1 mg/mL gelatin from porcine skin (Sigma-Aldrich). Gels were rinsed three times with 2.5% Triton X-100, followed by incubation for 12 h at 37 °C in 50 mM Tris-HCl (pH 7.5), 10 mM CaCl_2_, and 200 mM NaCl. Gels were stained with Coomassie Blue, and areas of gelatinolytic activity were visualised as transparent bands.

### Xenografts and metastasis experiments in mice

The Ethical Committee of the Consejo Superior de Investigaciones Científicas (Madrid, Spain) and Community of Madrid (PROEX 252/15) approved the protocols used for experimental work with animals. For liver homing assessment, mice were inoculated in spleen with 1×10^6^ KM12SM cells, and euthanised 48 h after inoculation. RNA was isolated from liver using TRIzol (Ambion, Carlsbad, CA, USA), retrotranscribed and 0.3 mg cDNA subjected to PCR with Taq DNA polymerase (Invitrogen) to amplify human GAPDH as a surrogate.^15^ As a control, a 20-cycle PCR amplification of murine β-actin was performed. For liver metastasis, Swiss nude mice (*n* = 6 per condition) were inoculated in the spleen with 1.5×10^6^ human KM12SM cells in 0.1 mL PBS in presence or absence of the indicated peptides (1 µg/mL). A day later, spleens were removed to avoid local growth. Peptide treatment started 2 days after inoculation and lasted for 2 weeks. Mice were inoculated intravenously with the indicated peptides (7 doses of 3 µg in 0.1 mL PBS). For lung metastasis, Balb/c mice were inoculated in the tail vein with 1×10^6^ mouse CT-26 cells in 0.1 mL PBS and treated intravenously with the peptide as indicated before. When signs of disease were visible, mice were euthanised, subjected to necropsy, and inspected for metastasis in liver or lungs. Asymptomatic mice were sacrificed at day 80 and inspected for metastasis in liver.

For glioblastoma xenografts, 10^7^ U251 cells were inoculated subcutaneously into the flanks of NSG mice. Eleven days after cell inoculation, when tumours reached a size of 100 mm^3^, mice were treated with D-D1 peptide (3 μg/100 μL of PBS) subcutaneously or with saline solution in a total of 7 doses during 15 days. Tumours were measured every 2–3 days.

### Statistical analyses

Data were analysed by one-way ANOVA followed by Tukey−Kramer multiple comparison test. Survival curves were plotted with Kaplan–Meier technique and compared with the log-rank test. The minimum acceptable level of significance was *p* < 0.05.

## Results

### Selection of cancer cell lines and design of the IL13Rα2 blocking peptide

**Fig. 1:**
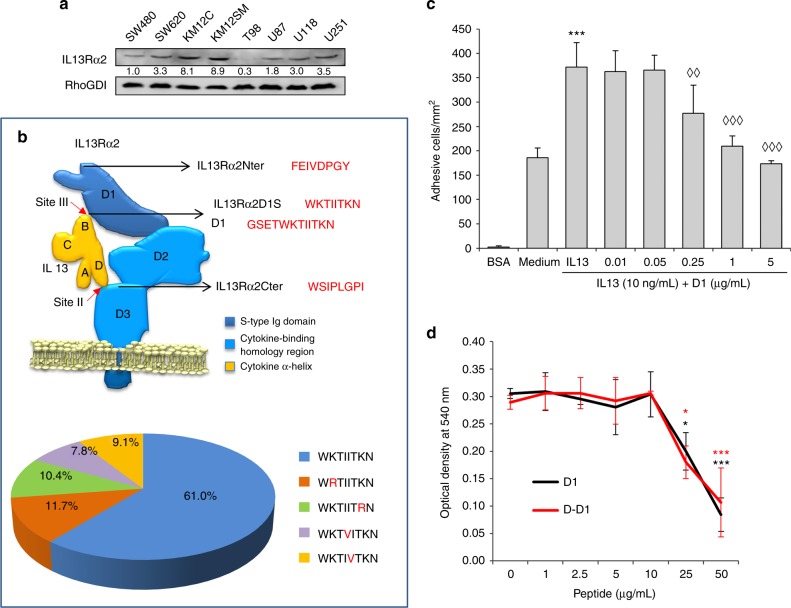
IL13Rα2 expression in colorectal cancer and glioblastomas. Identification of IL13Rα2 peptides able to inhibit IL13 binding. **a** Western blot analysis of IL13Rα2 expression in the indicated cell lines of colon cancer and glioblastoma. **b** Schematic representation of IL-13/IL13Rα2 binding domains indicating the different peptide sequences used for analysis. The D1 peptide motif was investigated in 77 mammalian species. Percentages of each sequence variant in mammalians were represented in a pie chart. **c** KM12SM cells treated with IL-13 (10 ng/mL) and the indicated concentrations of D1 peptide were subjected to adhesion assays in Matrigel. Cell adhesion was significantly enhanced by IL-13 (***p < 0.001). The presence of D1 peptide inhibited IL13-stimulated cell adhesion (◊◊*p* < 0.01; ◊◊◊*p* < 0.001). **d** The presence of D1 or D-D1 peptides significantly inhibited the interaction of IL-13 to immobilised IL13Rα2 by competition ELISA (*p < 0.05; ***p < 0.001)

First, we investigated the expression of the IL13Rα2 receptor in different colorectal and glioblastoma cancer cell lines (Fig. 1a). The receptor was present at different extent in all the cell lines tested, except glioblastoma T98 cells. Next, we designed an IL13Rα2 12-mer peptide (GSETWKTIITKN) (named D1) using the sequence that interacts directly with IL-13 in site III^24^ (Fig. 1b). These residues form a lineal sequence highly conserved between different mammalian species (Fig. 1b). Other IL13Rα2 interaction sites with IL-13 are conformational and require multiple residues in noncontiguous positions that are more difficult to mimic in a synthetic peptide. Different amounts of D1 peptide (from 10 ng/mL to 5 µg/mL) were tested to inhibit IL-13-mediated cell adhesion in metastatic KM12SM colorectal cancer cells (Fig. 1c). A progressive increase in the IL-13 blocking capacity was observed at the highest doses of the D1 peptide (1–5 µg/mL). As a balance between effectiveness and dose, we selected 1 µg/mL for the remaining experiments.

To study the peptide effect on IL-13 binding, we performed a competition binding experiment between the peptides and the recombinant IL-13 for the receptor. D1 and D-D1 peptides blocked IL-13 binding to IL13Rα2 starting at 10 µg/mL, with near complete inhibition at 50 µg/mL (Fig. 1d). Collectively, these results support a direct interaction between the peptide and IL-13 that blocks its binding capacity.

### IL13Rα2 D1 peptide inhibits the metastatic capacity of colorectal and glioblastoma cell lines

**Fig. 2:**
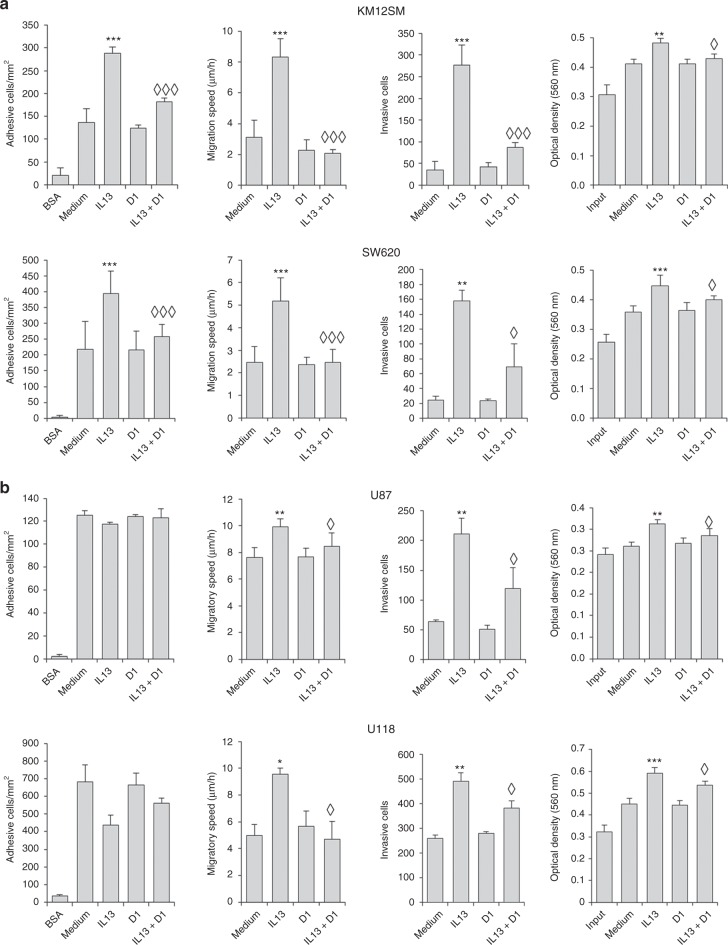
IL13Rα2 D1 peptide inhibits IL13-mediated cell migration, invasion and proliferation of colon cancer and glioblastoma cells. **a** Metastatic KM12SM and SW620 colon cancer cells or **b** U87 and U118 glioblastoma cells treated with IL-13 were subjected to cell adhesion, wound healing, cell invasion through Matrigel and MTT assays in the presence of the D1 peptide. Number of adhesive cells, effective migration speed, number of invasive cells or viable cells were significantly enhanced by IL-13 addition (*p< 0.05; **p < 0.01; ***p < 0.001). However, the D1 peptide significantly inhibited cell adhesion, migration invasion and proliferation triggered by IL-13 (◊*p* < 0.05; ◊◊◊*p* < 0.001)

Next, we examined the capacity of the D1 peptide to inhibit cell adhesion, migration, invasion and proliferation in two colorectal, KM12SM and SW620, and two glioblastoma cell lines, U87 and U118. In both colorectal cell lines, the addition of IL-13 provoked a clear increase in adhesion, migration and invasion, and, at a lesser extent, proliferation **(**Fig. 2a). The addition of D1 peptide to IL-13-treated cells caused a near complete inhibition of the IL-13 prometastatic effects in both colon cancer cell lines, being particularly effective in the inhibition of the invasiveness capacity (Fig. 2a). D1 peptide alone did not cause any effect. In glioblastoma cells, IL-13 efficiently promoted the invasion capacity. Migration and proliferation were induced, although at a lesser extent, and there was no effect on cell adhesion, suggesting the activation of different pathways to colorectal cancer (Fig. 2b). Regardless of the cell line, the D1 peptide abolished IL-13 effects on invasion, migration and proliferation in both glioblastoma cell lines. Collectively, these results indicate that the presence of the D1 peptide interferes with IL-13 protumourigenic properties, especially with the invasive capacity.

### Essential residues for the blocking of IL-13 binding to IL13Rα2

**Fig. 3:**
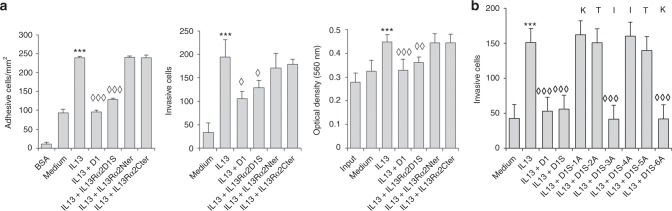
Sequence-specific blocking effect and alanine scanning of D1S peptide. **a** KM12SM cells were treated with IL-13 in the presence of two D1 peptides (long and short sequence) and two unrelated IL13Rα2 peptides and subjected to cell adhesion, invasion and MTT assays. Cell adhesion, invasion or proliferation triggered by IL13 (***p < 0.001) were inhibited by the presence of the indicated peptides (◊*p* < 0.05; ◊◊*p* < 0.01; ◊◊◊*p* < 0.001). **b** For testing alanine scanning, invasion assays of IL-13-treated KM12SM cells in the presence of the alanine-mutated D1S peptides were performed

We tested a shorter version of the D1 peptide (D1S) containing only the preserved core motif “WKTIITKN” to block adhesion, invasion and proliferation of KM12SM colorectal cancer cells. Blocking IL-13 with the peptide D1S caused similar effects to the longer 12 amino acid peptide (Fig. 3a). In contrast, two peptides from the IL13Rα2 N-terminus (FEIVDPGY) and C-terminus (WSIPLGPI) failed to cause any effect on cell adhesion, invasion and proliferation (Fig. 3a). Then, we performed an alanine scanning experiment for peptide WKTIITKN, where each residue was replaced by an alanine. Residues W and N were not included as they were invariable in all mammalian motif sequences. Control and mutated peptides were added to KM12SM cells together with IL-13. Replacement of ^84^I and ^87^K by Ala did not alter the invasion capacity of the D1 peptide, indicating that these two residues are not essential for the IL-13 binding (Fig. 3b). Interestingly, alterations of any of the other four residues caused a complete loss of the inhibitory properties. These results help to explain the strong conservation of this motif in the IL13Rα2 sequence.

### IL13Rα2 D1 peptide inhibited IL-13-mediated cell signalling in colorectal cancer and glioblastoma

**Fig. 4:**
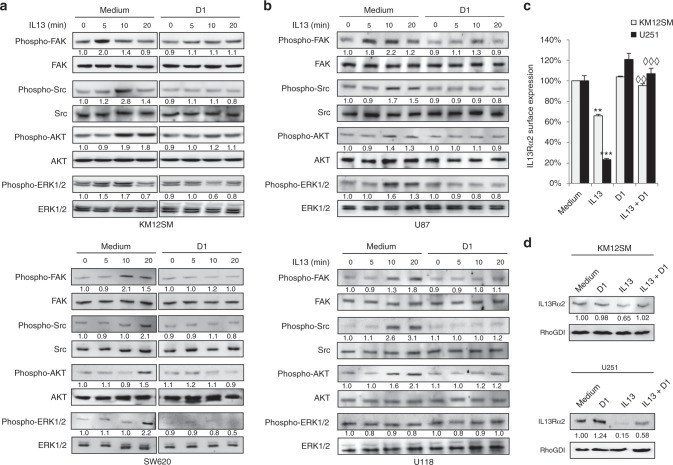
Molecular pathway analysis of cell signalling inhibition by D1 peptide. **a** KM12SM and SW620 colorectal cancer cell lines or **b** U87 and U118 glioblastoma cells were treated with IL-13 for the indicated times in serum-free DMEM in absence or presence of D1 peptide. Cell extracts were analysed by western blot with antibodies against FAK, JNK, ERK1/2, Src, AKT and their phosphorylated forms. The D1 peptide inhibited phosphorylation of IL-13 mediators in a time and cell type-dependent mode. **c** KM12SM and U251 cells were treated with IL13 and/or D1 peptide for 1 h and analysed by flow cytometry to detect cell surface IL13Rα2 or (**d**) by western blot for total IL13Rα2. Whereas IL13 significantly promoted receptor internalisation (**p < 0.01; ***p < 0.001), D1 significantly inhibited IL13-triggered internalisation (◊◊*p* < 0.01; ◊◊◊*p* < 0.001). RhoGDI was used as loading control

Next, we investigated the effect of the D1 peptide on the downstream IL-13/ IL13Rα2 signalling in colorectal and glioblastoma cell lines. In metastatic KM12SM colorectal cells, IL-13 provoked a rapid activation (5 min) of phospho-FAK and phospho-ERK1/2, followed by activation of phospho-Src and phospho-AKT at 10 min. Addition of D1 peptide caused a marked reduction of IL-13 signalling, with a significant decrease in the phosphorylation of the four downstream mediators (FAK, Src, AKT and ERK1/2) at different times, between 5 and 20 min post-IL13 (Fig. 4a). In U87 glioblastoma cells, the results were similar. After incubation with IL-13 there was a fast activation of FAK, followed by Src, AKT and ERK1/2, which was fully blocked after addition of the D1 peptide to the cells (Fig. 4b). To confirm these results, we examined two additional cell lines from colorectal cancer (SW620) and glioblastoma (U118). With some minor differences in the activation times, D1 peptide abrogated activation of FAK, Src, AKT and ERK1/2 in these cell lines (Fig. 4a, b). The capacity of IL-13 to induce matrix metalloproteases (MMPs) in IL13Rα2-positive pancreatic tumour cells to promote metastasis has been described.^5^ Here, we investigated the presence of MMPs in IL13Rα2-positive cells using gelatinolytic zymography, to detect the active forms, and western blot. Although we did not observe changes in MMP2 and MMP9 by zymography, there was an induction of MT1-MMP in U251 cells but not in KM12SM cells (Supplementary Fig. S1). D1 peptide inhibited the expression of MT1-MMP, which may contribute to reduce the invasive capacity of glioblastoma cells.

On the other hand, we studied IL13Rα2 internalisation as a mechanism of control for signalling regulation.^15^ IL13Rα2 internalisation promoted by IL-13 was significantly reduced after treatment with D1 peptide (Fig. 4c). The D1 peptide also prevented the IL13Rα2 degradation caused by IL-13 binding and internalisation in KM12SM and U251 cells (Fig. 4d). Taken together, these results suggest the capacity of the D1 peptide to inhibit IL-13-mediated cell signalling through FAK, Src, AKT and ERK1/2 in colorectal cancer and glioblastoma cell lines and the induction of MT1-MMP in glioblastoma cells. Moreover, D1 peptide treatment reduced receptor internalisation by endosomal recycling and/or degradation.

### IL13Rα2 D1 peptide partially inhibits IL-13 binding to IL13Rα1

**Fig. 5:**
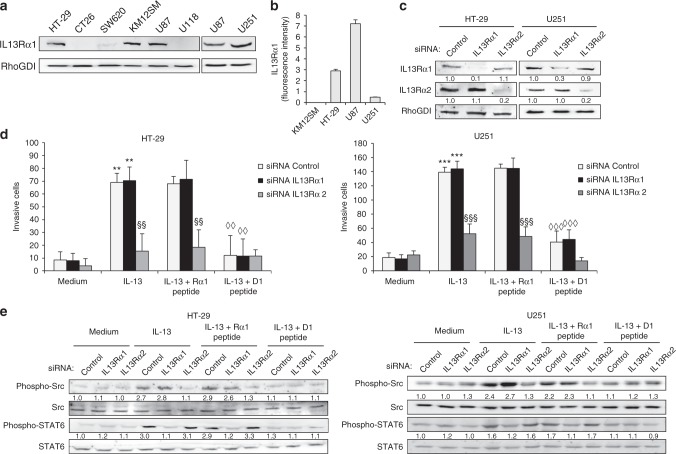
IL13Rα2-derived peptide inhibits IL13Rα1 and IL13Rα2-mediated signalling. **a** Cell lines were lysed and analysed by western blot to detect IL13Rα1 expression. RhoGDI was used as loading control. **b** IL13Rα1-positive cells were subjected to flow cytometry to detect IL13Rα1 expression on the cell surface. **c** HT-29 colon cancer and glioblastoma U251 cells were transfected with IL13Rα1 and IL13Rα2 siRNAs or control. At 48 h, transfected cells were lysed and subjected to western blot to confirm the silencing of expression. RhoGDI was used as loading control. **d** The same transfectants were used for IL-13 triggered invasion in the presence of Rα1 or D1 peptides. IL-13-mediated cell invasion (**p < 0.01; ***p < 0.001) was inhibited after IL13Rα2 silencing (§§*p* < 0.01; §§§*p* < 0.001) or by the addition of D1 peptide (◊◊*p* < 0.01; ◊◊◊*p* < 0.001). **e** Cells treated as in (**d**) were analysed by western blot to detect Src, phospho-Src, STAT6 and phospho-STAT6. Total Src and STAT6 were used as loading controls

Although with dramatic differences in affinity,^24^ IL-13 uses the same residues to bind IL13Rα1 and IL13Rα2 in site III of the receptor. Therefore, since the D1 peptide could also block the binding of IL13 to the more widespread receptor IL13Rα1, we investigated the presence of IL13Rα1 in different colorectal cancer and glioblastoma cell lines. We observed expression of IL13Rα1 in the colorectal cancer cell line HT-29 and the glioblastoma cell lines U251 and U87 in both cellular extracts (Fig. 5a) and plasma membrane (Fig. 5b). Next, we performed transient siRNA silencing of both receptors in the three cell lines (Fig. 5c, Supplementary Fig. S2A). As anticipated, IL13Rα2-silencing, but not IL13Rα1 silencing, abolished cell response to IL-13 and suppressed the invasive capacity of HT-29, U251 and U87 cell lines mediated by IL-13 (Fig. 5d, Supplementary Fig. S2B). We also examined the blocking specificity of the D1 peptide with respect to the receptor. We carried out different strategies to discriminate the IL-13 binding inhibition to IL13Rα1 and IL13Rα2.

Initially, we prepared a peptide from IL13Rα1, equivalent to D1, named Rα1 (KQDKKIAPE), to test its functional activity on the invasive capacity of the receptor-silenced cell lines (Fig. 5d, Supplementary Fig. S2B). Whereas D1 peptide inhibited IL-13-mediated invasion in IL13Rα1-silenced or control cells in both types of cancer, Rα1 peptide did not. So, the Rα1 peptide did not block IL-13 binding to cancer cells. Therefore, despite selecting IL-13 binding residues to both receptors, IL-13 interaction with both receptors and peptide-mediated effects are different and not interchangeable. Finally, we investigated the capacity of IL13Rα2 D1 peptide to inhibit the activation of the Src and STAT6 pathways mediated by IL-13 binding to the IL13Rα1/IL-4Rα heterodimer. HT-29, U251 and U87 cell lines siRNA-silenced for IL13Rα1 and IL13Rα2 receptors were treated with IL-13 and analysed for STAT6 and Src activation in the presence or not of Rα1 and D1 peptides (Fig. 5e, Supplementary Fig. S2C). Whereas IL13Rα1-silencing caused a substantial inhibition of STAT6 phosphorylation, IL13Rα2-silencing decreased the levels of STAT6 activation only after D1 treatment. Therefore, the D1 peptide significantly inhibits STAT6 and Src phosphorylation, particularly in HT-29 cells. Finally, we prepared two IL-13 mutants: E13Y and I15A, which have a very low affinity for IL13Rα1, maintaining the high affinity for IL13Rα2. Interestingly, both IL-13 mutants showed the same proinvasive activity in colorectal cancer and glioblastoma cells than the wild-type IL-13 (Supplementary Fig S3A). Conversely, the D1 peptide was able to block the proinvasive capacity of both mutants independently of the affinity for the receptor. Additionally, strong inhibition of STAT6 signalling was observed for the IL-13 mutants, the remaining marginal activation was also abolished by the D1 peptide (Supplementary Fig S3B). These results strongly support that D1 peptide neutralises IL-13 binding to both receptors, although at different extent. These results might be clinically relevant as IL13Rα1 is involved in different IL-13-associated pathologies.

### The D1 enantiomer preserves functionality and improves peptide stability in blood

A major limitation in the use of peptide-based therapies is the poor half-life of peptides in biological fluids. Peptides composed of natural amino acids are target of multiple proteases present in the blood. To overcome this limitation we prepared an enantiomer version of the D1 peptide consisting of D amino acids, named D-D1, which is known to increase peptide stability.^25,26^ It was demonstrated by a major resistance to proteolytic digestion (data not shown). To investigate any potential impact of this modification in the biological activity of the peptide, KM12SM cancer cells were incubated with IL-13 plus D1 and D-D1 peptides. No differences on the adhesion, invasion and proliferation effects were observed between both peptides (Supplementary Fig. S4). These results support the use of the D-D1 enantiomer for in vivo experiments.

### IL13Rα2 peptide inhibits colorectal cancer metastasis and glioblastoma growth

**Fig. 6:**
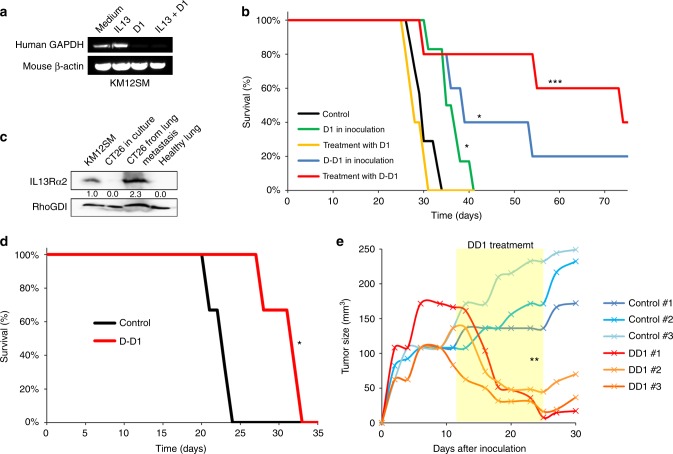
D-D1 enantiomer increases mice survival to colorectal cancer metastasis and inhibits glioblastoma xenografts. **a** For liver homing, KM12SM cells were treated with the IL13 and/or D1 peptide and inoculated in spleen of nude mice. 2 days after inoculation, RNA was isolated from liver and subjected to RT-PCR to amplify human GAPDH. Mouse β-actin was used as loading control. **b** Kaplan–Meier survival results for nude mice intrasplenically inoculated with metastatic KM12SM cells treated with the peptides only (D1 in inoculation) or treated with seven doses of peptide (treatment with D1) administered intravenously. Mice survival was significantly enhanced after treatment with the peptides (*p < 0.05; ***p < 0.001). **c** Mouse CT-26 cells were isolated from Balb/c lung metastases, lysed and analysed for IL13Rα2 expression by western blot and compared to extracts from cultured CT-26 cells. KM12SM lysates were used as positive control for IL13Rα2 expression and RhoGDI as loading control. **d** Kaplan−Meier survival results for Balb/c mice inoculated with CT-26 cells in tail vein, and treated with seven doses of D-D1 peptide. Mice survival was significantly longer after peptide treatment (*p < 0.05). **e** U251 cells were inoculated subcutaneously. 11 days after inoculation, mice were treated with D-D1 for 2 weeks (yellow panel). Tumour growth is represented for both control and D-D1-treated mice. Treatment with D-D1 significantly inhibited tumour growth (**p < 0.01).

First, we investigated the effect of the D1 peptide on the liver homing capacity of the KM12SM cells. Swiss nude mice were inoculated in the spleen with metastatic KM12SM cells treated with IL-13, D1 peptide or both. Mice were euthanised at 48 h after inoculation and RNA isolated from mice livers. KM12SM metastatic cells pretreated with the D1 peptide, with or without IL-13, failed to colonise the liver, as no human GAPDH amplification was detected by PCR (Fig. 6a).

Next, we evaluated the capacity of D1 and the enantiomer D-D1 peptides to increase mouse survival to metastasis (Fig. 6b). We followed two experimental approaches: either KM12SM cells were inoculated together with the peptide (1 µg/mL) or mice were treated with the peptide (7 doses of 3 µg in 0.1 mL PBS) starting 48 h after cell injection. For the D1 peptide, there was a modest increase in mice survival when cells were preincubated with the peptide, but no effect was observed after treatment (Fig. 6b). In contrast, mice inoculated with KM12SM cells and treated with D-D1 peptide showed a remarkable increase in survival, with 40% of the mice surviving the experimental endpoint without metastatic lesions in liver. Mice inoculated with cells preincubated with the peptide also displayed a significant increase in survival, with 20% of the mice surviving without metastatic presence (Fig. 6b). Then, we tested the peptide protection capacity using a very aggressive, undifferentiated, mouse CT-26 colorectal cancer cell line. Mouse CT-26 cells gain IL13Rα2 expression after homing and exposure to IL-13 in lung metastasis (Fig. 6c). This induction occurs likely through NKT cells, which in turn induce IL13Rα2 expression in the lungs after colonisation, within the strategy of cancer cells to evade immune response.^27^ Treatment with the D-D1 peptide induced a significant survival increase in mice with lung metastasis (31 vs. 23 days, 35% increase in the mean survival time). This partial effectiveness might be due to the late “de novo” induction of IL13Rα2 expression in CT26 cells (Fig. 6d). Collectively, these results demonstrate the capacity of the D-D1 peptide to protect against liver and, partially, lung metastasis, in different metastatic conditions.

Finally, we tested the capacity of the D-D1 peptide to inhibit tumour growth in U251 glioblastoma xenografts. Tumours were left to grow for 10 days before starting the treatment. D-D1 peptide administration caused a significant growth arrest accompanied by a regression in the tumour size (Fig. 6e). These results suggest that the peptide administration might be effective also after tumour implantation.

## Discussion

Here, we provide multiple evidences that an IL13Rα2 peptide constitutes a simple and effective strategy for the inhibition of the IL-13 prometastatic capacity in colorectal cancer and glioblastoma growth: (a) the peptide significantly inhibited the proliferation, migration and invasion properties of metastatic colorectal cancer and glioblastoma cell lines in a very efficient way; (b) there was a marked reduction of the IL-13 signalling capacity through IL13Rα2 to activate the FAK, PI3K/AKT and Src pathways; (c) MT1-MMP induction was decreased; (d) the D1 peptide significantly inhibited IL13Rα1-mediated STAT6 activation; (e) the peptide induced “in vivo” protection against human and mouse metastatic cells in liver and lung metastasis, respectively and (f) glioblastoma xenograft growth was arrested in the presence of the peptide. Since overexpression of IL13Rα2 receptor has been reported in triple-negative breast cancer^28^ and ovarian cancer,^10^ among others, the use of this peptide might have a broad application in different types of cancer.

The IL-13/IL13Rα2 molecular recognition displays one of the highest affinities in nature (~500 pM).^24^ As the D1 peptide is ten times smaller (1377 Da) than the IL-13 (15,816 Da), the ability of the peptide to compete with the IL-13 binding suggests an inhibitory effect based on a large excess of the low-affinity peptide to displace the high-affinity interaction between IL-13 and IL13Rα2. The unusual conservation of the 8-mer motif (^81^WKTIITKN^88^) among 77 analysed mammalian species suggests a high specificity and a critical dependence from this motif in the IL13Rα2 binding receptor. This motif contains the palindromic sequence KTIITK. The role and significance of protein palindromic motifs remain unclear. They have been associated to low complexity protein regions and have shown a greater propensity to form α-helical structures.^29^ However, in this case, the IL13Rα2 site III contacts are forming an extended antiparallel β-sheet interaction.^24^ Peptides have low immunogenicity compared to antibodies or proteins; the risk of eliciting an antipeptide immune response in the treated individuals is quite low. Here, the risk is even lower, since the D1 peptide sequence is very well preserved among many different species.

D1 peptide not only inhibited the Src and PI3/AKT pathways, but also significantly suppressed the induction of MT1-MMP in glioblastoma cells. In glioblastoma, MT1-MMP produces tumour growth and local invasion of the extracellular matrix.^30^ Therefore, inhibition of MT1-MMP expression induced by IL-13 might play a key role to control tumour-cell invasion. Remarkably, D1 not only blocked IL-13 binding to IL13Rα2, but also inhibited IL13Rα1-mediated STAT6 activation in both cancer types. The effectiveness of the D1 peptide to inhibit signalling by blocking IL-13 binding to both receptors might be beneficial from a clinical point of view. A monoclonal antibody that neutralises IL-13 binding to both receptors has shown clinical benefits in patients with asthma with acceptable safety and tolerability for the patients.^31^ Moreover, 16% of GBM tumours are highly positive for IL13Rα1 and 40% are strongly positive for IL13Rα2.^32^ Coexpression of both markers results in a poorer outcome of the patients.^32^ Also, abundant expression of IL13Rα1 is associated to poor prognosis in invasive breast cancer.^33^ Although metastatic cancer cells usually do not express IL13Rα1 or IL-4Rα^1^, or they do it in small amounts^1^, the D1 peptide effect on IL13Rα1-activation should not be detrimental but probably synergistic in metastatic cancer cells.

The IL-13/IL13Rα2 binding complex is internalised after binding, as indicated by the association of IL13Rα2 to the scaffold protein FAM120A and intracellular transport molecules.^15^ Our results suggest a reduction of signalling associated to a decrease in IL13Rα2 internalisation. In some cases, internalisation of the activated receptor (i.e. EGFR) and endocytic trafficking enables specific signalling pathways from intracellular sites.^34^ In this regard, the regulation of IL13Rα2 expression by mutant variant EGFRvIII in glioblastoma cells might suggest some association between both receptors.^3^ It is unclear whether IL13Rα2 trafficking and recycling is critical for IL-13 signalling as it occurs for EGFR and its ligands. However, our results point out that this could be part of the mechanism of action of the D1 peptide that seems to inhibit the ligand-mediated internalisation. On the other hand, targeting IL13Rα2 expression on immune cells of the tumour microenvironment contributes to the restoration of the immunosurveillance mechanisms caused by the loss of TGFβ1 production.^27^ Therefore, blocking IL13Rα2 might reduce mouse mortality through two different mechanisms, either a direct effect on the tumoural cells or an indirect effect on myeloid cells (CD11b^high^/Gr-1^intermediate^) for restoration of cellular immunity. This indirect effect remains to be tested.

Therapeutic peptides are generally used to interfere with natural receptors, kinases or other functional proteins. However, there are not many successful examples of peptides with therapeutic properties in cancer.^26,35^ A major limitation of peptides is their short life in biological fluids in vivo. To increase peptide life-length and potency, the use of D-amino acids is among the most reliable strategies.^25,26^ D-peptides present multiple advantages: easy to synthesise, accelerated lead identification, enhanced specificity and binding of the hindered targets without many of the disadvantages of other therapeutic molecules (expensive manufacturing, off-target side effects, etc.). Other peptide delivery systems that might be tested would include peptide-loaded nanoparticles for an efficient and controlled-release peptide delivery.

In conclusion, D1 peptide inhibits IL-13 binding to IL13Rα2, and partially IL13Rα1, ligand-mediated internalisation and signalling, reducing metastatic colonisation. This approach opens novel strategies to develop a promising therapeutic candidate for the treatment of advanced metastatic colorectal cancer. Further improvements could be based on peptide sequence variants with improved affinity, different doses, treatment duration, delivery route, etc. Further experiments will have to validate the effectiveness of the peptide in already established tumours. Given our results with glioblastoma cells and the abundant expression of IL13Rα2 in other tumours, we can envisage that this strategy could be useful for targeting glioblastoma and, probably, other pathologies. However, we need to test the capacity of the peptide to overcome the blood brain barrier for glioblastoma therapy. Finally, it might be interesting to evaluate its application to common diseases where IL-13 signalling through both receptors plays a critical role, such as asthma and/or atopic dermatitis.^20^

## Electronic supplementary material

Supplementary Figures

## Availability of data and materials

Original data and materials are available upon request. No data sets were generated in this manuscript.
