# Supplementary material for: An IL13Rα2 peptide exhibits therapeutic activity against metastatic colorectal cancer
Source: Br J Cancer. 2018 Oct 15;119(8):940–9. doi: 10.1038/s41416-018-0259-7 (PMC6203792; doi:10.1038/s41416-018-0259-7)
Supplement: Supplementary file 1 — Supplementary Figures [file 41416_2018_259_MOESM1_ESM.pptx]

## Slide 1
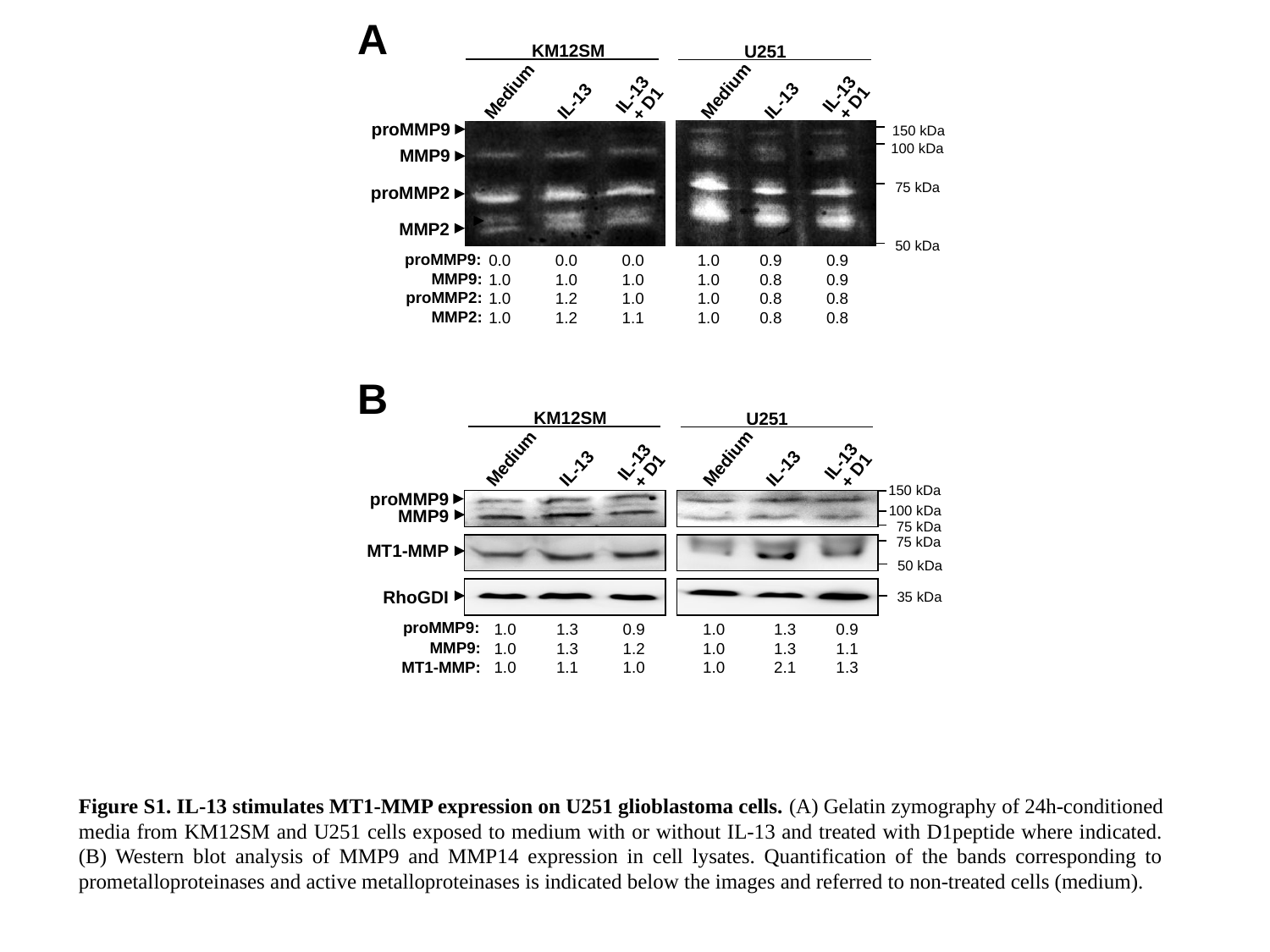

A
KM12SM
U251
Medium
Medium
IL-13 + D1
IL-13 + D1
IL-13
IL-13
proMMP9
150 kDa
100 kDa
 75 kDa
 50 kDa
MMP9
proMMP2
MMP2
proMMP9:
 0.0 0.0 0.0 1.0 0.9 0.9
 1.0 1.0 1.0 1.0 0.8 0.9
 1.0 1.2 1.0 1.0 0.8 0.8
 1.0 1.2 1.1 1.0 0.8 0.8
MMP9:
proMMP2:
MMP2:
B
KM12SM
U251
Medium
Medium
IL-13 + D1
IL-13 + D1
IL-13
IL-13
150 kDa
proMMP9
100 kDa
MMP9
 75 kDa
 75 kDa
MT1-MMP
 50 kDa
RhoGDI
 35 kDa
proMMP9:
 1.0 1.3 0.9 1.0 1.3 0.9
 1.0 1.3 1.2 1.0 1.3 1.1
 1.0 1.1 1.0 1.0 2.1 1.3
MMP9:
MT1-MMP:
Figure S1. IL-13 stimulates MT1-MMP expression on U251 glioblastoma cells. (A) Gelatin zymography of 24h-conditioned media from KM12SM and U251 cells exposed to medium with or without IL-13 and treated with D1peptide where indicated. (B) Western blot analysis of MMP9 and MMP14 expression in cell lysates. Quantification of the bands corresponding to prometalloproteinases and active metalloproteinases is indicated below the images and referred to non-treated cells (medium).

## Slide 2
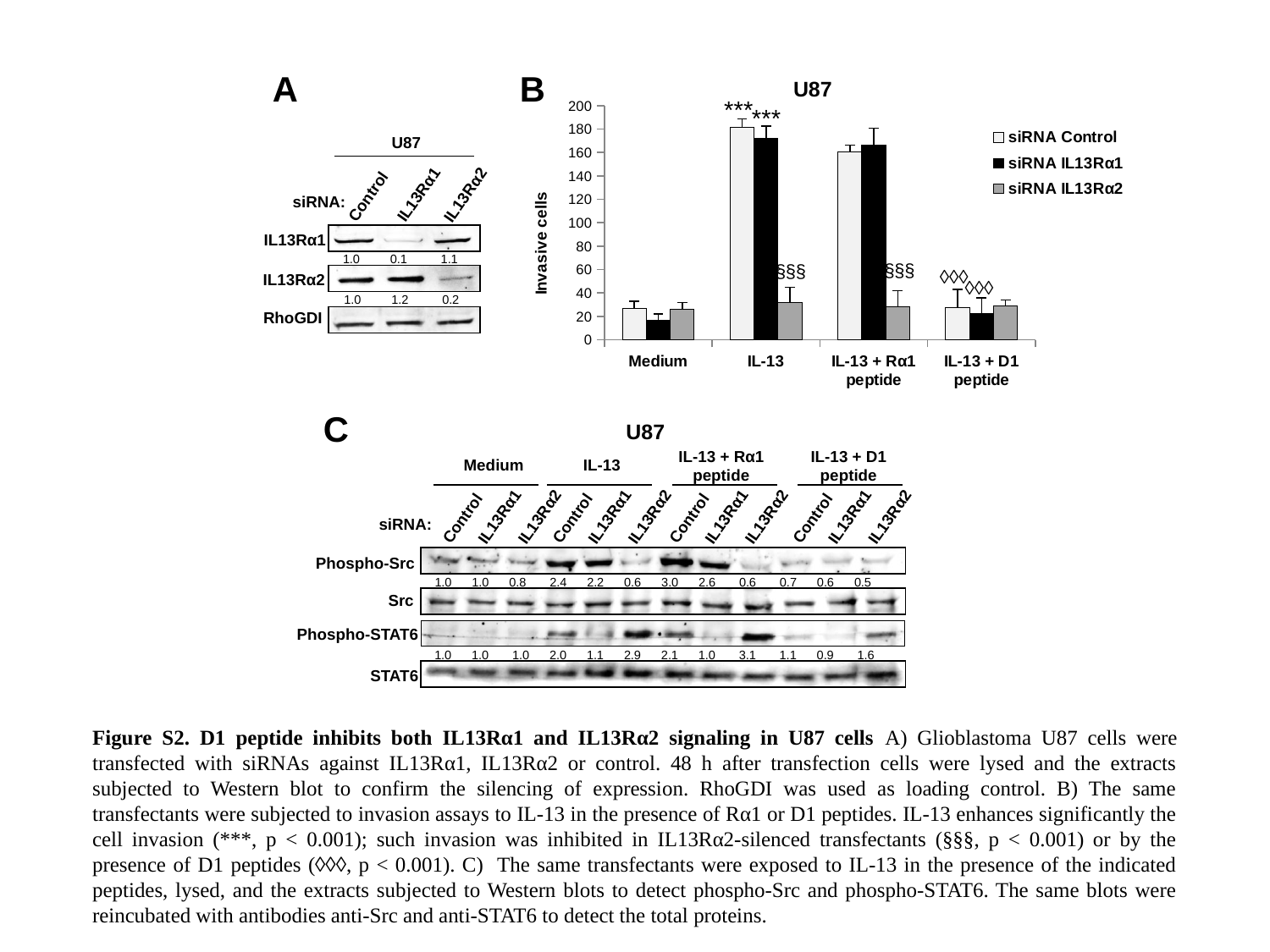

### Chart
| Category | siRNA Control | siRNA IL13Rα1 | siRNA IL13Rα2 |
|---|---|---|---|
| Medium | 26.5 | 16.5 | 26.0 |
| IL-13 | 181.5 | 172.0 | 31.5 |
| IL-13 + Rα1 peptide | 160.5 | 166.0 | 28.5 |
| IL-13 + D1 peptide | 27.5 | 22.5 | 29.0 |B
A
U87
***
***
U87
Control
IL13Rα1
IL13Rα2
siRNA:
IL13Rα1
1.0 0.1 1.1
§§§
§§§
◊◊◊
IL13Rα2
◊◊◊
1.0 1.2 0.2
RhoGDI
C
U87
IL-13 + Rα1 peptide
IL-13 + D1 peptide
Medium
IL-13
Control
Control
Control
Control
IL13Rα1
IL13Rα1
IL13Rα1
IL13Rα1
IL13Rα2
IL13Rα2
IL13Rα2
IL13Rα2
siRNA:
Phospho-Src
1.0 1.0 0.8 2.4 2.2 0.6 3.0 2.6 0.6 0.7 0.6 0.5
Src
Phospho-STAT6
 1.0 1.0 1.0 2.0 1.1 2.9 2.1 1.0 3.1 1.1 0.9 1.6
STAT6
Figure S2. D1 peptide inhibits both IL13Rα1 and IL13Rα2 signaling in U87 cells A) Glioblastoma U87 cells were transfected with siRNAs against IL13Rα1, IL13Rα2 or control. 48 h after transfection cells were lysed and the extracts subjected to Western blot to confirm the silencing of expression. RhoGDI was used as loading control. B) The same transfectants were subjected to invasion assays to IL-13 in the presence of Rα1 or D1 peptides. IL-13 enhances significantly the cell invasion (***, p < 0.001); such invasion was inhibited in IL13Rα2-silenced transfectants (§§§, p < 0.001) or by the presence of D1 peptides (◊◊◊, p < 0.001). C) The same transfectants were exposed to IL-13 in the presence of the indicated peptides, lysed, and the extracts subjected to Western blots to detect phospho-Src and phospho-STAT6. The same blots were reincubated with antibodies anti-Src and anti-STAT6 to detect the total proteins.

## Slide 3
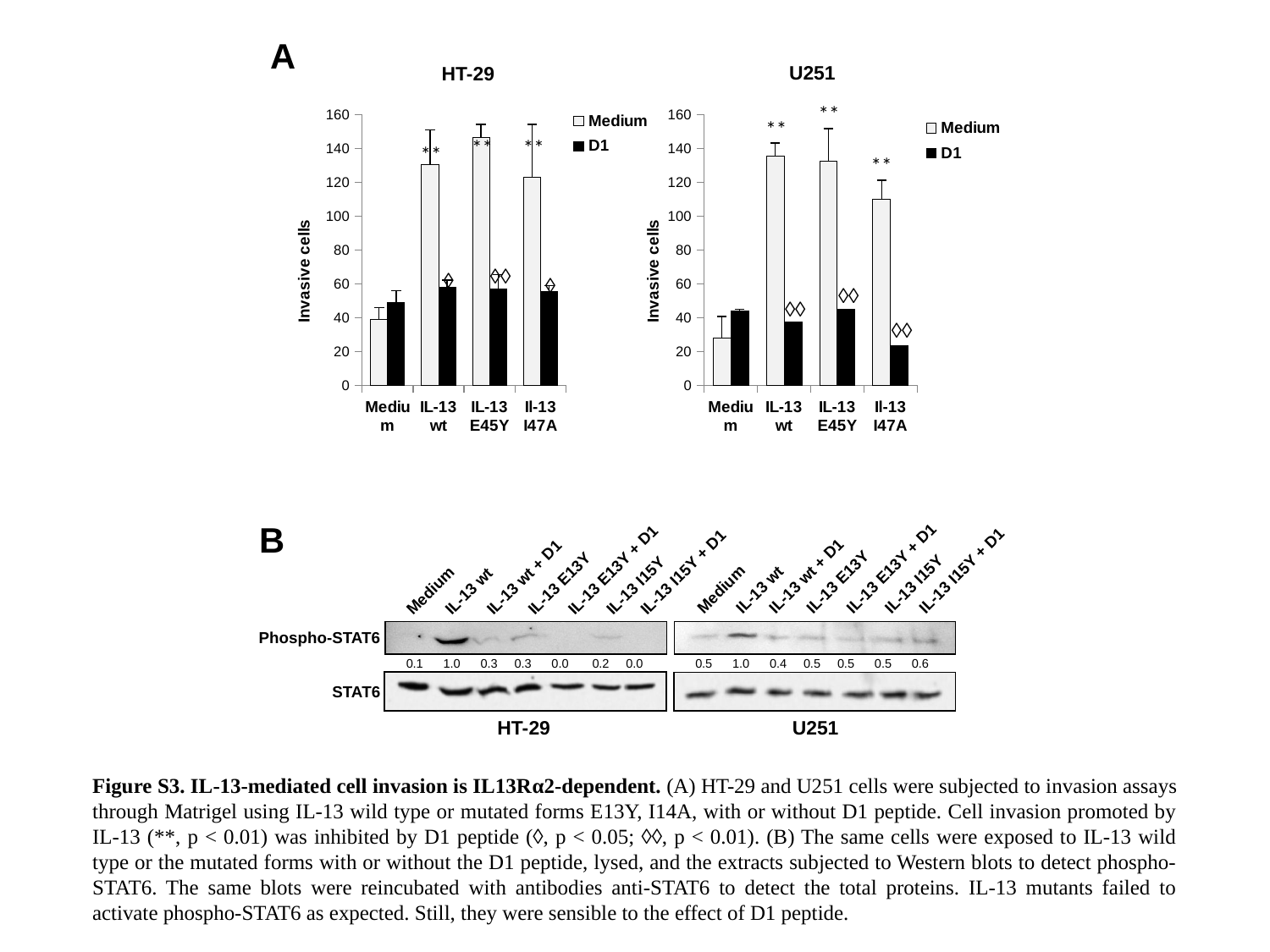

A
U251
HT-29
### Chart
| Category | Medium | D1 |
|---|---|---|
| Medium | 39.0 | 49.0 |
| IL-13 wt | 130.5 | 58.0 |
| IL-13 E45Y | 146.5 | 57.0 |
| Il-13 I47A | 123.0 | 55.5 |
### Chart
| Category | Medium | D1 |
|---|---|---|
| Medium | 28.0 | 44.0 |
| IL-13 wt | 135.5 | 37.5 |
| IL-13 E45Y | 132.5 | 45.0 |
| Il-13 I47A | 110.0 | 23.5 |**
**
**
◊◊
◊
◊
◊◊
◊◊
◊◊
B
IL-13 E13Y + D1
IL-13 E13Y + D1
IL-13 I15Y
IL-13 I15Y + D1
IL-13 I15Y
IL-13 I15Y + D1
Medium
IL-13 wt
IL-13 wt + D1
IL-13 E13Y
Medium
IL-13 wt
IL-13 wt + D1
IL-13 E13Y
Phospho-STAT6
0.1 1.0 0.3 0.3 0.0 0.2 0.0
0.5 1.0 0.4 0.5 0.5 0.5 0.6
STAT6
HT-29
U251
Figure S3. IL-13-mediated cell invasion is IL13Rα2-dependent. (A) HT-29 and U251 cells were subjected to invasion assays through Matrigel using IL-13 wild type or mutated forms E13Y, I14A, with or without D1 peptide. Cell invasion promoted by IL-13 (**, p < 0.01) was inhibited by D1 peptide (◊, p < 0.05; ◊◊, p < 0.01). (B) The same cells were exposed to IL-13 wild type or the mutated forms with or without the D1 peptide, lysed, and the extracts subjected to Western blots to detect phospho-STAT6. The same blots were reincubated with antibodies anti-STAT6 to detect the total proteins. IL-13 mutants failed to activate phospho-STAT6 as expected. Still, they were sensible to the effect of D1 peptide.

## Slide 4
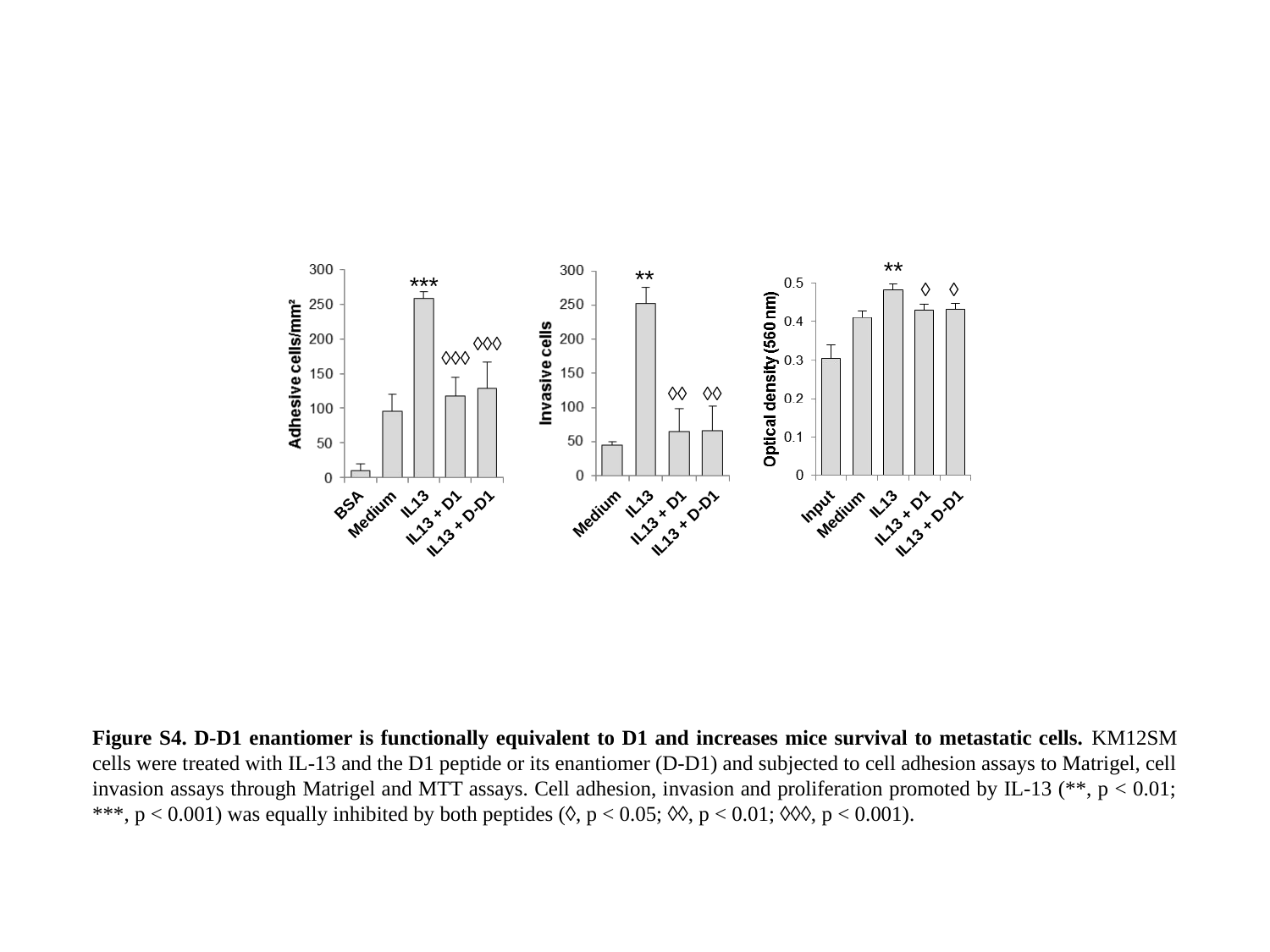

**
**
***
◊
◊
◊◊◊
◊◊◊
◊◊
◊◊
Medium
IL13
IL13 + D1
IL13 + D-D1
BSA
Medium
IL13
IL13 + D1
IL13 + D-D1
Input
Medium
IL13
IL13 + D1
IL13 + D-D1
Figure S4. D-D1 enantiomer is functionally equivalent to D1 and increases mice survival to metastatic cells. KM12SM cells were treated with IL-13 and the D1 peptide or its enantiomer (D-D1) and subjected to cell adhesion assays to Matrigel, cell invasion assays through Matrigel and MTT assays. Cell adhesion, invasion and proliferation promoted by IL-13 (**, p < 0.01; ***, p < 0.001) was equally inhibited by both peptides (◊, p < 0.05; ◊◊, p < 0.01; ◊◊◊, p < 0.001).
